# Effects of Pyrolysis Temperature and Acid-Base Pre-Treatment on the Synthesis of Biochar-Based Slow-Release Selenium Fertilizer and Its Release in Soil

**DOI:** 10.3390/ma17040879

**Published:** 2024-02-14

**Authors:** Jun Chu, Suikai Wang, Jie Yu, Yuting Gao, Zhenya Tang, Qiliang Yang

**Affiliations:** 1 Faculty of Modern Agricultural Engineering, Kunming University of Science and Technology, Kunming 650500, China; chujun1218@163.com (J.C.); sj18087149853@163.com (S.W.); yujie20011013@163.com (J.Y.); gaoyuting@stu.kust.edu.cn (Y.G.); 2 Yunnan Provincial Field Scientific Observation and Research Station on Water-Soil-Crop System in Seasonal Arid Region, Kunming University of Science and Technology, Kunming 650500, China; 3 Yunnan Provincial Key Laboratory of High-Efficiency Water Use and Green Production of Characteristic Crops in Universities, Kunming University of Science and Technology, Kunming 650500, China

**Keywords:** selenium, biochar, polyethyleneimine, modified biochar, selenium release rate

## Abstract

Plant-derived selenium is an important source of selenium (Se) for humans, which, however, has been restricted by a low content of Se in soil. Traditional Se fertilizers have tended to result in low selenium utilization. Thus, it was necessary to develop a new slow-release material to control Se fertilizer release. In this study, biochar pyrolyzed at 300 °C and 800 °C was cross-linked with polyethyleneimine (PEI) after being treated with HNO_3_ or NaOH (which were labeled Acid-W300, Acid-W800, Alkali-W300, and Alkali-W800). The results showed that the maximum adsorption capacities of Acid-W300, Alkali-W300, Acid-W800, and Alkali-W800 were 329.16 mg/g, 321.93 mg/g, 315.04 mg/g, and 344.33 mg/g, respectively. Among them, Acid-W800 and Alkali-W800 were mainly imine- and amide-bonded with SO_3_^2−^, while Acid-W300 and Alkali-W300 were loaded with SO_3_^2−^ by forming the C–Se bonding as well as through imine- and amide-bonding. The release of four biochar-based selenium fertilizers in the red soil and brown soil extracts conformed to the pseudo-second-order kinetic model. The release rate and release amount of four biochar-based selenium fertilizers in the red soil extract were higher than those in the brown soil extract. Alkali-W800-Se had a higher proportion of Se-exchangeable release, accounting for 87.5% of the total loaded selenium, while Acid-W300-Se had the lowest proportion at 62.2%. However, the Se releases of Alkali-W800-Se were more than 42.49% and 37.67% of the total Se-loading capacity during 5 days of continuous red soil extraction and brown soil extraction, respectively. Acid-W300-Se released less than 20% of the total Se-loading capacity. Thus, Acid-W300-Se was the recommended slow-release Se fertilizer in red soil and brown soil.

## 1. Introduction

Selenium (Se) is one of the essential trace elements for maintaining homeostasis in humans [1]. Selenium deficiency can lead to a series of cardiovascular diseases, and severe selenium deficiency can also lead to Keshan disease and Kashin–Beck disease [2]. Food is the main source of selenium intake for the human body [1]. The content and availability of soil Se have a decisive impact on the Se content of food materials, including crops. Studies have shown that more than two-thirds of China’s soil has low-selenium or selenium-deficient conditions (selenium-deficient: <0.125 mg/kg; low selenium: 0.125 mg/kg–0.175 mg/kg) [3,4,5]. This results in a per capita selenium intake in China (20–30 μg/d) that is well below the national recommended amount (50–250 μg/d) by the Chinese Nutrition Society [6]. With the improvement in living standards and the enhancement of dietary diversity in recent years, Kashin–Beck or Keshan diseases caused by selenium deficiency have been initially controlled. The other diseases, however, caused by insufficient selenium intake, are an upward trend. For example, Tsuji reported that the high incidence of lung cancer in Xuanwei, China, is related to the local low-selenium blood status. Insufficient selenium intake still poses a threat to people’s health in Se-deficient areas [6,7].

The application of Se fertilizers is the main measure used to increase the selenium content of plants in selenium-deficient areas. However, the direct application of a selenium fertilizer can easily lead to the loss of selenium or the fixation of selenium by soil. The slow and controlled release of such fertilizers has attracted wide attention in the field of fertilizers in recent years. A slow-and-controlled-release fertilizer can not only improve the fertilizer’s utilization rate but also effectively reduce fertilizer loss [8,9]. Carbon-based slow-release fertilizers have become a research hotspot because their materials are cheap, easy to obtain, and they can increase the soil’s carbon content. Biochar produced by anaerobic pyrolysis has received a lot of attention recently. Studies have shown that biochar could improve the availability of Se in soil and crops [8,9,10,11]. Additionally, biochar generally has larger porosity, which can improve soil quality and reduce nutrient loss. Therefore, the development of biochar-based fertilizers has become one of the important methods for improving soil, promoting crop quality, and reducing environmental risks. However, studies have shown that biochar’s ability to load anions is limited due to its electronegative surface [12]. Thus, it is necessary to modify the biochar to improve the loading capacity of Se.

Polyethyleneimine (PEI), which is composed of numerous primary and secondary amine groups, exhibits excellent complexation abilities for heavy metals, anions, etc., due to the strong chemical affinity between those ions or anions and N-containing functional groups. Studies have shown that PEI-modified biochar or other carbonaceous adsorbents can effectively improve the adsorption capacity of HCrO_4_^−^ [12] and PO_4_^3−^ [13] anions. However, the research on PEI-modified biochar mainly focuses on sewage treatment to remove heavy metals and other harmful substances in sewage [13,14]. Those studies mainly concern the loading capacity of modified biochar with pollutants and its ability to be recycled. As a slow-and-controlled-release fertilizer carrier, modified biochar is not only required to have a high loading capacity for selenium but must also ensure that it can be slowly released in the soil to achieve a slow and controlled release. Whether PEI-modified biochar Se fertilizers can be released stably in the soil to meet the needs of crop growth needs to be analyzed further [14]. Therefore, research on the loading and releasing characteristics of modified biochar and its releasing rule of selenium in soil solutions has important theoretical value and practical significance for improving the availability of selenium in soil and preventing diseases caused by selenium deficiency [14,15,16].

## 2. Materials and Methods

### 2.1. Preparation of Biochar and PEI-Modified Biochar

The eucalyptus wood chips (Qiqun, Chongqing, China) were weighed and placed in the Muffle furnace before anaerobic pyrolysis at 300 °C and 800 °C for 4 h with nitrogen gas at a flow rate of 1.5 L/min, which were labeled as W300 and W800, respectively.

Before being modified with polyethyleneimine (PEI), the biochar was pretreated with acid and alkali [16,17,18]. Briefly, the flasks with 20 g of W300 or W800 were added with 200 mL of an 8% (wt.%) HNO_3_ or 3M KOH solution and then stirred at 40 °C at 160 rpm for 3 h. The biochars were then washed with deionized water to a pH of 7.0. The biochars were dried in the oven at 80 °C for 12 h. The treated biochars were added into the conical flask with 100 mL of a 10% (*w*/*v*) PEI/methanol solution and then shaken for 24 h at 70 °C at 160 rpm. After that, 200 mL of a 1% (*w*/*v*) glutaraldehyde solution was added into the conical flask before shaking for 30 min at 70 °C at 160 rpm. The modified biochar was washed 3 times with deionized water before being air-dried. The four PEI-modified biochars were labeled as Acid-W300, Acid-W800, Alkali-W300, and Alkali-W800.

### 2.2. PEI-Modified Biochar-Based Se Fertilizer Loading Experiment

#### 2.2.1. Batch Adsorption

A total of 5 mg of Acid-W300, Alkali-W300, Acid-W800, and Alkali-W800 biochar were added into a conical flask with 5 mL of 0.1 g Se/L, 0.3 g Se/L, 0.5 g Se/L, 0.8 g Se/L, 1 g Se/L, 1.5 g Se/L, or 2.0 g Se/L of the NaSeO_3_ solution with a solution pH of 7.0, respectively. The conical flasks were shaken in 25 °C conditions at 200 r/min for 24 h. An amount of 2 mL of the samples was collected with a syringe from the conical flasks, and then the remaining selenium in the solution was analyzed by the atomic fluorescence method after being filtered with 0.22 μm of the filter membrane. The batch adsorption experimental data were fitted with the Langmuir adsorption isotherm (1), Freundlich adsorption isotherm (2), and Temkin adsorption isotherm (3) [18]:

*q_e_* = *K_L_* × *q_max_* × *C_e_*/(1 + *K_L_* × *C_e_*) Langmuir (1)

*q_e_* = *K_F_* × *C_e_^n^* Freundlich (2)

*q_e_* = *RT* × *ln*(*A* × *C_e_*)/*b* Temkin (3)

where the “*q_e_*” is the equilibrium adsorption capacity (mg/g); the “*q_max_*” in Formula (1) is the theoretical maximum adsorption capacity (mg/g); the “*C_e_*” in (1)–(3) are the concentrations in the solution at equilibrium; the “*K_L_*” (L/mg) and “*K_F_*” (mg^(1−n)^ × L^n^/g) are the Langmuir and Freundlich adsorption coefficients, respectively; the “*n*” in (2) indicates preferential adsorption as *n* > 1 and the multilayer adsorption as *n* < 1. A (L/mg) and *b* (J∗g/mg) are Temkin adsorption constants.

#### 2.2.2. Adsorption Kinetics

30 mg of Acid-W300, Alkali-W300, Acid-W800, and Alkali-W800 biochar were added into a conical flask with 30 mL of 1 g Se/L of the Na_2_SeO_3_ solution with a solution pH of 7.0, respectively. The flasks were shaken in 25 °C conditions at 160 r/min. An amount of 1.5 mL of the samples were collected with a syringe at 5 min, 10 min, 20 min, 40 min, 1 h, 1.5 h, 2 h, 3 h, 4 h, 6 h, 10 h, 14 h, 24 h, and 36 h, respectively. The concentration of selenium in the supernatant was determined after filtering with a 0.22 μm filter membrane. The equilibrium adsorption experimental data were fitted to SeO_3_^2−^ by the pseudo-first-order kinetic model (4) and pseudo-second-order kinetic model (5):

(4) $$\frac{q_{t}}{q_{a}}=1-e^{-k_{1}t}\;\mathrm{pseudo-first-order}\;\mathrm{kinetic}\;\mathrm{model}$$

(5) $$\frac{q_{t}}{q_{a}}=\frac{k_{2}t\;}{1+{\;k}_{2}t}\;\mathrm{pseudo-second-order}\;\mathrm{kinetic}\;\mathrm{model}$$

where the “*q_t_*” is the adsorption capacity of Se at *t* time (mg/g); the “*q_a_*” is the maximum adsorption capacity of Se; the “*k*_1_” is the pseudo-first-order kinetic rate constant (min^−^^1^); and the “*k*_2_” is the pseudo-second-order kinetic rate constant (g/mg/min).

#### 2.2.3. The Loading Capacity of Selenite

A total of 200 mg of Acid-W300, Alkali-W300, Acid-W800, and Alkali-W800 biochar were added to 200 mL of 1.5 g/L of the Na_2_SeO_3_ solution with a solution pH of 7.0, respectively. The flasks were shaken in 25 °C conditions for 24 h at 160 r/min. Afterward, the mixed solution and the supernatant were collected and diluted 100-fold using 1 M nitric acid solution. Meanwhile, the original Se solution was diluted with the same method. After that, the Se concentration in the resulting solution was measured. The Se-loaded biochar, including Acid-W300, Alkali-W300, Acid-W800, and Alkali-W800, were labeled as Acid-W300-Se, Alkali-W300-Se, Acid-W800-Se, and Alkali-W800-Se, respectively. The loading capacity of biochar for the Se was calculated by the following Formula (6) [19]:

*Loading capacity* (mg/g) = *A* × (*C_pre_* − *C_sup_*) × *V*/*M*_0_ (6)

where the “*A*” is the dilution ratio; the “*C_pre_*” and “*C_sup_*^”^ are, respectively, the concentration of Se in the original solution and the diluted solution after the reaction, respectively; the “*V*” is the volume of the solution; and the “*M*_0_” is the adsorbent carbon mass.

### 2.3. PEI-Modified Biochar Se Fertilizer Releasing Experiment

#### 2.3.1. The Maximum Exchange-Releasing Potential of PEI-Modified Biochar Se Fertilizer

A total of 3 mg of Acid-W300-Se, Alkali-W300-Se, Acid-W800-Se, or Alkali-W800-Se, produced in Section 2.2.3, were added into a conical flask with 15 mL of the Mehlich-3 soil effective state extract solution, respectively. The flasks were shaken in 25 °C conditions with 200 r/min for 1 h, and then 2 mL samples were taken to pass through the 0.22 μm filter membrane for analysis. Se release and the release potential of biochar were calculated by the following formula:

*Se-releasing capacity* (mg/g) = *C_e_* × *V*/*M* (7)

*Se releasing potential* (%) = *Se releasing capacity*/*Se-loading capacity* × 100% (8)

where the “*C_e_*” is the concentration of Se in the solution; the “*V*” is the volume of the solution; and the “*M*” is the mass of PEI-modified biochar Se fertilizer.

#### 2.3.2. Release Character of Se in Red and Brown Soil

The red soil and brown soil collected from the experimental base of the Kunming University of Science and Technology, China, were chosen for evaluating the release characteristics of slow-and-controlled-release fertilizers in red soil and brown soil. The red soils or brown soils were passed through a 60-mesh screen before addition to ultra-pure water with a ratio of water/soil 2.5:1. The red soil and brown soil solutions were filtered with a 0.45 μm filter membrane after ultrasonic extraction for 1 h and labeled as a red soil extract or brown soil extract, respectively.

A total of 20 mg of Acid-W300-Se, Alkali-W300-Se, Acid-W800-Se, and Alkali-W800-Se were added into the flasks with 50 mL of the red soil extract and brown soil extract, respectively. The flasks were shaken in 25 °C conditions at 200 rpm. A total of 1 mL of the samples were collected to pass through a 0.22 μm filter membrane for determining Se at 10 min, 20 min, 30 min, 40 min, 1 h, 2 h, 4 h, 6 h, 8 h, 11 h, 14 h, and 24 h. The remaining samples were centrifuged, and the supernatant was poured off. A total of 50 mL of red or brown soil extract was added again, and the supernatant was centrifuged through a 0.22 μm filter membrane by oscillating at 160 rpm for 24 h at 25 °C to be tested. The above work was repeated until the fifth day. The desorption kinetic data were fitted using the pseudo-first-order kinetic (4) and pseudo-second-order kinetic models (5).

### 2.4. Characterization of Biochar and Determination of Selenium

The C, H, N, O, and S element contents of the biochars were determined using an element analyzer (Vario MicroCube, Elementar Company, Frankfurt, Germany). The surface functional groups of biochar were determined by Fourier transform infrared spectroscopy (is50 FT-IR, Thermo Fisher Scientific, Waltham, MA, USA) and an X-ray photoelectron spectrometer (Escalab 250Xi, Thermo Fisher Scientific, MA, USA). The Se content was measured by an atomic fluorescence spectrophotometer (AFS-620, Beijing Rayleigh Analytical Instrument Corp, Beijing, China). The Zeta potential was measured using a ZETASIZER 3000 HSA system (Malvern Panalytical, Shanghai, China). The sample was scanned using a TM3030 scanning electron microscope (SEM) (Hitachi, Tokyo, Japan).

### 2.5. Analysis Statics

Batch adsorption and adsorption kinetics data were plotted and fitted in Origin 2018; XPS data were split using XPS peak41 and plotted using Origin 2018. The differences in the Se-loading and releasing amounts were analyzed using SPSS 18 with Duncan’s test, with a *p* < 0.05, for testing the adsorption and desorption amounts of each modified biochar.

## 3. Results and Discussion

### 3.1. Characterization of Biochar before and after Modification

The pyrolysis temperature is an important factor affecting the physicochemical properties of biochar. Before modification, the eucalyptus wood chips and biochar were mainly composed of C (47.00%–91.73%), H (1.90%–7.20%), and O (5.90%–40.50%) elements (Table 1). The contents of N (0.20%–0.40%) and S (0.02%–0.15%) were less than 1%. The C and N elements in W800 were higher than those in W300 or wood chips, while the contents of H and O elements were opposite. The atomic ratios of H/C, O/C, and (O + N)/C all showed a decreasing trend, indicating that the polarity of biochar decreased with increasing pyrolysis temperature [20], which was consistent with other studies [20,21,22].

After modification, the C content in W300 (67.9%) decreased to 49.16% in Acid-W300 and 56.2% in Akali-W300, respectively. The C content in W800 was 91.73%, 80.4% in Acid-W800, and 76.9% in Akali-W800, respectively. However, the N or O elements content in PEI-modified biochar greatly increased compared with the corresponding original biochar. It is worth noting that the N content in Acid-W300 was 7.8 folds than that in Acid-W800. The N content in Akali-W300 (6.3%) was higher than that in Akali-W800 (5.78%). This indicated that the biochar produced in low temperatures contained more functional groups, which can improve the loading capacity of PEI in biochar [13]. 

**Figure 1:**
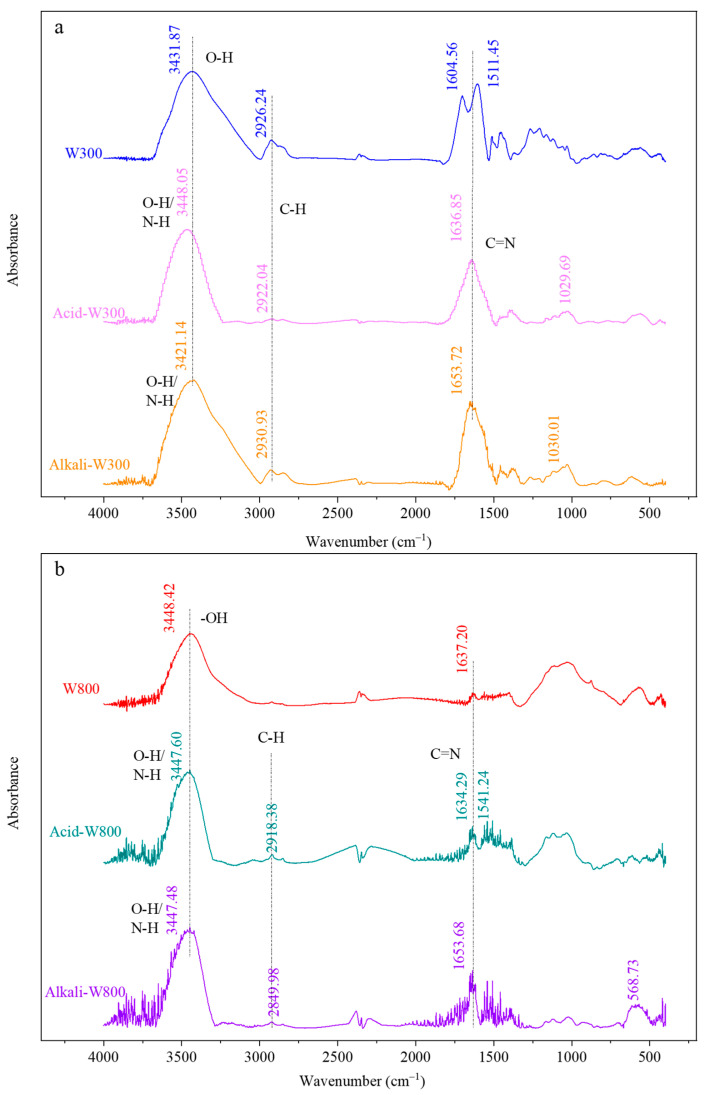
FIRT of biochar at 300 °C before and after PEI modification (**a**) and at 800 °C before and after PEI modification (**b**). The descriptions of the treatments are in the footnote of Table 1.

The FT-IR of the original biochar and the PEI-modified biochar showed that a new peak appeared at near 1626.85 cm^−1^ in Acid-W300, Alkali-W300, Acid-W800, and Alkali-W800 (Figure 1). This peak presented a C=N stretching vibration absorption peak, indicating that the Schiff-base reaction occurred between the carbonyl group and the amino group on glutaraldehyde.

**Figure 2:**
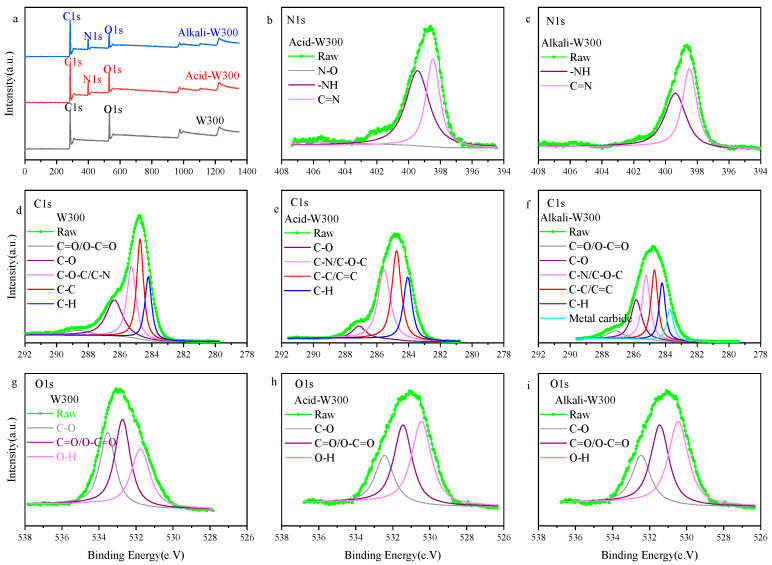
XPS spectra of PEI-modified biochar at 300 °C before and after modification. The (**a**) is the XPS spectrum before and after PEI modification at 300 °C; (**d**,**g**) are the C1s and O1s sub-peaks of biochar at 300 °C before PEI modification, respectively. (**b**,**e**,**h**) are the sub-peak diagrams of N1s, C1s, and O1s of Acid-W300, respectively. (**c**,**f**,**i**) are sub-peak diagrams of N1s, C1s, and O1s of Alkali-W800, respectively. The descriptions of the treatments are in the footnote of Table 1.

**Figure 3:**
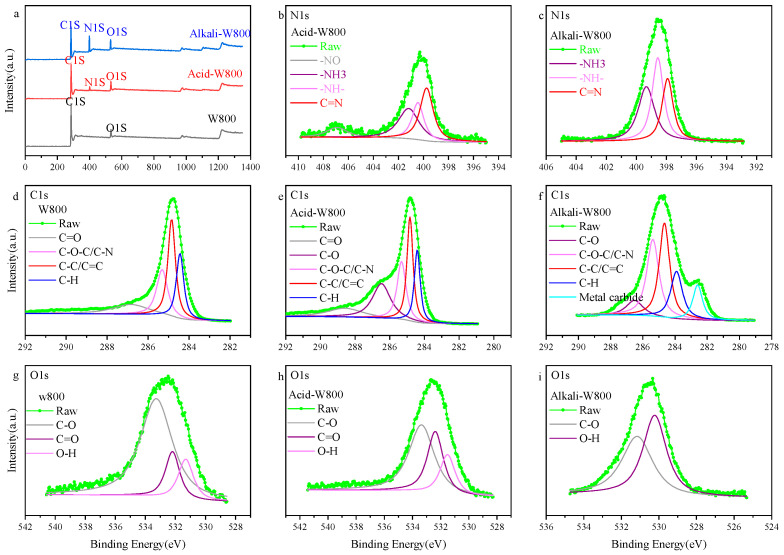
XPS energy spectra before and after PEI modification by pyrolytic biochar at 800 °C. The (**a**) is the XPS spectrum before and after PEI modification at 800 °C; (**d**,**g**) are the C1s and O1s sub-peaks before PEI modification, respectively. (**b**,**e**,**h**) are the sub-peak diagrams of N1s, C1s, and O1s of Acid-W800, respectively. (**c**,**f**,**i**) are sub-peak diagrams of N1s, C1s, and O1s of Alkali-W800, respectively. The descriptions of the treatments are in the footnote of Table 1.

XPS analysis was used to determine the surface functional group characteristics of biochar and modified biochar. As shown in Figure 2a and Figure 3a, a significant N_1_s peak appeared in Acid-W300, Alkali-W300, Acid-W800, and Alkali-W800. The N-spectrograph revealed two main forms of N (-NH: 399.36 eV, and C=N: 398.59 eV bonds) in Acid-W300 (Figure 2b) and Alkali-W300 (Figure 2c), and three main forms of N (-NH_3_/-NH_2_: 400.38 eV, -NH: 399.36 eV, and C=N: 398.59 eV) in Acid-W800 (Figure 3b) and Alkali-W800 (Figure 3c). Additionally, a small amount of N-O (404.2 eV) was found in Acid-W300 and Acid-W800. The C-O-C bond was mainly present at 285.34 eV. After modification, the area of functional groups at 285.34 eV of biochar accounted for an increase in the total area of carbon-containing functional groups compared with that of the original biochar (Figure 2e,f and Figure 3e,f). This was the result of the superposition of the C-N and C-O-C bonds, which was consistent with the FTIR (Figure 1). Additionally, the Zeta potential (Table S1) showed that the surface of the original biochars was in the negative charge with the lowest Zeta potential in W800 (−43.11 mV). The SEM (Figure S1a,b) also showed that W800 exhibited more pillar-like and honeycomb-like pores than that in W300. After modification, the Zeta potential of all four PEI-modified biochars turned to a positive charge, with the highest zeta potential in Alkali-W800 (43.76 mV), as well as the biochar surface becoming rough and irregular (Figure S1b,c,e,f). These results suggested that polyethyleneimine had been cross-linking into biochar.

### 3.2. Loading Characteristics of Modified Biochar on Sodium Selenite

The loading of biochar on SeO_3_^2−^ is affected by various external factors and its structure. This paper focuses on the effects of the initial concentration of SeO_3_^2−^ (0.1 mg/mL–1.5 mg/mL) and the contact time on the loading of SeO_3_^2−^ in the PEI-modified biochar. The adsorption equilibriums of Acid-W300 and Acid-W800 were reached when the initial concentration of SeO_3_^2−^ was at 1 mg/mL and that of Alkali-W800 was at 1.5 mg/mL (Figure S2a). This indicated that Alkali-W800 had better adsorption properties than other biochars.

**Figure 4:**
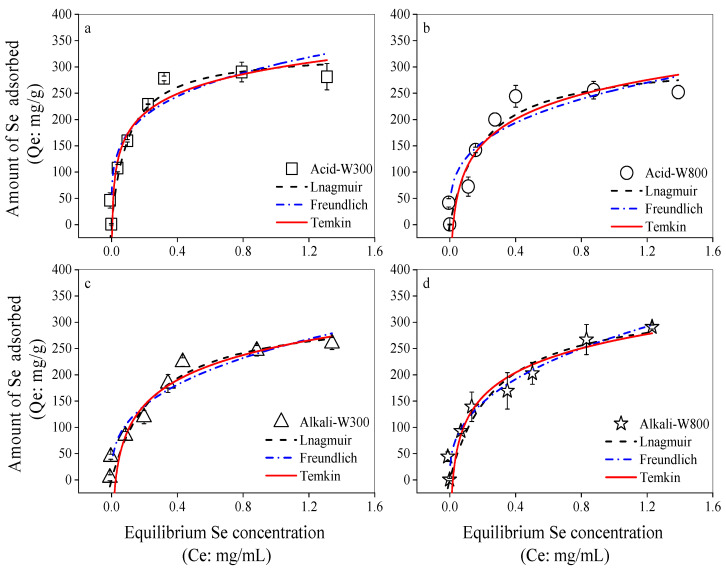
Isotherm adsorption curves of Acid-W300 (**a**), Acid-W800 (**b**), Alkali-W300 (**c**), and Alkali-W800 (**d**) with Langmuir, Freundlich, and Temkin models.

To clarify the adsorption mechanism, the Langmuir, Freundlich, and Temkin models were used to fit the loading data of equilibrium [23] (Figure 4, Table S2). The results showed that the Temkin isothermal adsorption models could well fit the adsorption data of Acid-W300 (R^2^ > 0.931, Table S2) and Alkali-W300 (R^2^ > 0.938, Table S2). The Freundlich isothermal adsorption models could well fit the adsorption data of Alkali -W800 (R^2^ > 0.960, Table S2). The Langmuir isothermal adsorption could well fit the Acid-W800 (R^2^ > 0.898, Table S2). This indicated that the adsorption process of SeO_3_^2−^ by the Acid-W300, Alkali-W300, and Alkali-W800 was multilayer adsorption, which was similar to the adsorption of Cr(Ⅵ) by PEI-modified biochar [24]. However, the adsorption of Acid-W800 to SeO_3_^2−^ was mainly on the surface of biochar.

**Figure 5:**
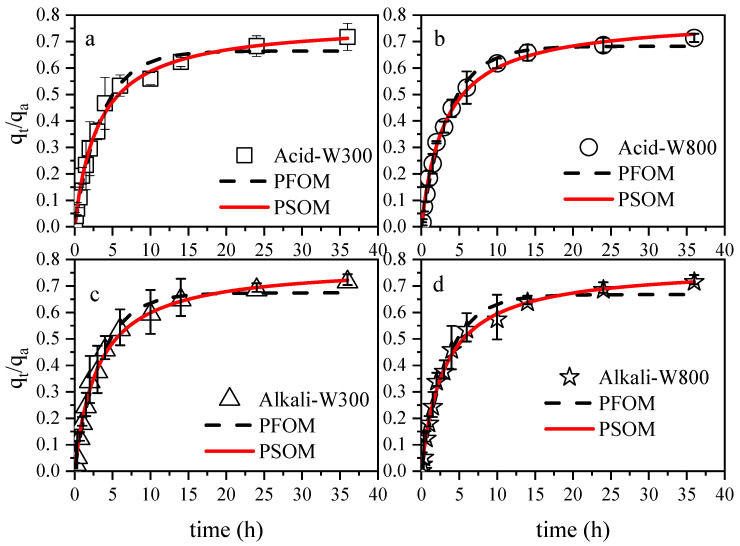
Kinetic adsorption curves of Acid-W300 (**a**), Acid-W800 (**b**), Alkali-W300 (**c**), and Alkali-W800 (**d**) with PFOM and PSOM models.

Furthermore, the influence of contact time on the adsorption and loading performance of SeO_3_^2−^ was studied. The SeO_3_^2−^-loading capacity rose rapidly in the first 6 h, and the loading capacity reached equilibrium at about 14 h (Figure S2b). To infer the adsorption mechanism of the adsorbent on SeO_3_^2−^, the pseudo-first-order, pseudo-second-order, and Elovich kinetic models were used for fitting data (Figure 5). The results showed that the pseudo-second-order kinetic model has higher correlation coefficients for the four adsorbents (Table S3). This indicated that the adsorption process of PEI-modified biochar on SeO_3_^2−^ was dominated by chemisorption. The surface of PEI-modified biochar was rich in functional groups, such as hydroxyl, amino, secondary amino, and amide groups, which had strong reactions and adsorption activity. Those functional groups could form imine bonds and amide bonds with SeO_3_^2−^ [15,24,25,26].

**Scheme 1:**
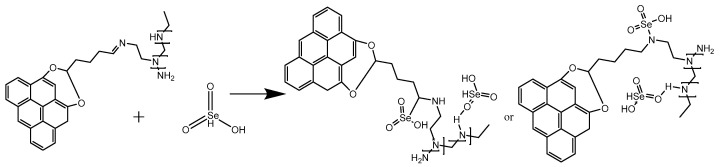
Possible reacting mechanism of PEI-modified biochar and SeO_3_^2−^.

**Figure 6:**
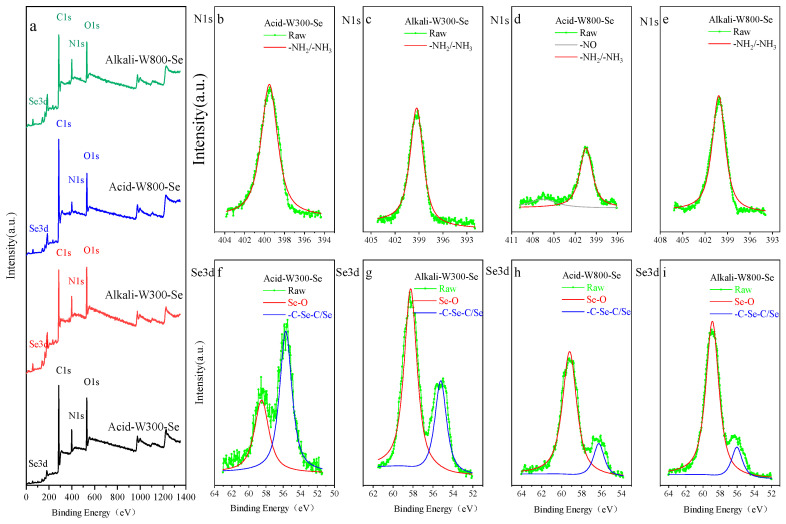
XPS spectra of PEI-modified biochar after SeO_3_^2−^ adsorption. (**a**) is the full XPS spectrum of PEI-modified biochar after SeO_3_^2−^ adsorption; (**b**–**e**) are the N1s sub-peaks of Acid-W300, Alkali-W300, Acid-W800, and Alkali-W800 after SeO_3_^2−^ adsorption, respectively. (**f**–**i**) are the Se3d sub-peaks of Acid-W300, Alkali-W300, Acid-W800, and Alkali-W800 after SeO_3_^2−^ adsorption, respectively.

Additionally, XPS showed that the Se peaks appeared after the adsorption of Se (Figure 3a, Figure 4a and Figure 6). This indicated that the Se had been loaded onto PEI-modified biochar. Two forms of Se, including Se-O (58.38 eV) and -C-Se-C/Se (55.64 eV), were shown in the modified biochar (Figure 6f–i). This indicated that the modified biochar could load Se through chemical reactions, except for imine- and amide-bonding in PEI-modified biochar. Interestingly, except for the -NO (406.43 eV) bond and -NH_2_/-NH_3_ (400.48 eV) in Acid-W800-Se, the N elements in the rest of the PEI-modified biochar were only in the forms of -NH_2_/-NH_3_ (400.48 eV) after the SeO_3_^2−^ adsorption. The C=N bond before adsorption (Figure 2b,c and Figure 3b,c) was not found after adsorption (Figure 6b–e). This indicated that the reaction occurred during the adsorption process to break the C=N bond. We speculate that a chemical reaction (Scheme 1) may have occurred at the C=N bond to form C-N-Se-O bonds or C-Se bonds [27].

From the perspective of each treatment, the selenium loaded in Acid-W800-Se and Alkali-W800-Se was mainly in the forms of Se-O, accounting for 85.4% and 82.7% of the total loaded selenium, respectively. The C-Se/Se bond in Alkali-W300-Se accounted for 32.3% of the total Se. However, the ratio of the C-Se bond in Acid-W300-Se was as high as 62.5% of the total loaded Se. This result might be related to the fact that biochar produced at low temperatures has more functional groups, such as C=N and C=C [1].

### 3.3. Release Characteristics of Modified Biochar-Based Selenium in Red and Brown Soils

As mentioned above, the modified biochar can reach the maximum adsorption capacity when the initial concentration of SeO_3_^2−^ is about 1.5 g/L and the contact time is 14 h. Thus, 200 mg of Acid-W300, Alkali-W300, Acid-W800, and Alkali-W800 biochar were added into 200 mL of 1.5 g of the Se/L Na_2_SeO_3_ solution before shaking in 25 °C conditions for 24 h at 160 r/min. The biochars were washed with deionized water three times and dried for the release characteristic test of the PEI-modified biochar-based selenium fertilizer. The results were consistent with the isothermal adsorption that the loading capacity of Acid-W300-Se, Alkali-W300-Se, Acid-W800-Se, and Alkali-W800-Se were 287.1 mg/g, 278.8 mg/g, 247.8 mg/g, and 314.1 mg/g, respectively (Table 2).

The Mehlich-3 soil-effective state extraction solution (0.2 mol/L CH_3_COOH, 0.25 mol/L NH_4_NO_3_, 0.015 mol/L NH_4_F, 0.013 mol/L HNO_3_, 0.001 mol/L EDTA) was used to verify the maximum exchangeable Se release amount of the four slow-release fertilizers [28,29]. The maximum exchangeable Se release amount of Alkali-W800-Se (275.0 mg/g) was the highest, which was significantly higher than that of Acid-W800-Se (176.7 mg/g). The maximum exchangeable Se release amount of Alkali-W300-Se and Acid-W300-Se were 198.9 mg/g and 178.6 mg/g, respectively. Similarly, the highest maximum exchangeable Se release rate was in Alkali-W800-Se (87.5%), followed by Alkali-W300-Se (71.3%), Acid-W800-Se (71.1%), and Acid-W300-Se (62.2%) [30,31,32]. The maximum exchangeable Se release rates of all four PEI-modified biochar-based seleniums were more than 50%. This indicated that the four PEI-modified biochar seleniums could be used as an effective selenium fertilizer [33,34,35]. The significantly high maximum exchangeable Se release rates in Alkali-W800-Se, Alkali-W300-Se, and Acid-W800-Se were related to Se in those mainly bonded in the forms of SeO_3_^2−^ through imine and amide bonds (Figure 6g–i). However, except for the imine and amide bonds with SeO_3_^2−^, a large proportion of Se in Acid-W300-Se was in the form of C-Se. This leads to the Se in Alkali-W800-Se and Acid-W800-Se being more easily exchanged and released into a solution [31,35,36,37].

**Figure 7:**
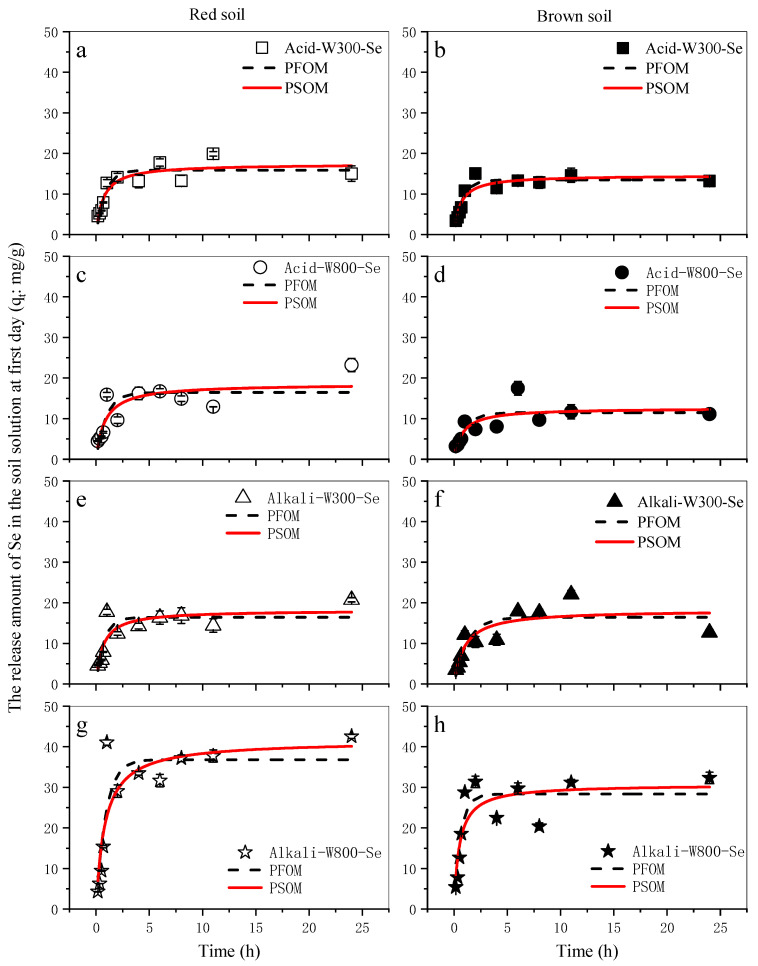
The Se release of Acid-W300-Se ((**a**) in the red soil extract and (**b**) in the brown soil extract), Acid-W800-Se ((**c**) in the red soil extract and (**d**) in the brown soil extract), Alkali-W300-Se ((**e**) in the red soil extract and (**f**) in the brown soil extract), and Alkali-W800-Se ((**g**) in the red soil extract and (**h**) in the brown soil extract) in red and brown soil solutions.

To evaluate the release kinetics in red soil and brown soil, the four Se fertilizers, including Acid-W300-Se, Alkali-W300-Se, Acid-W800-Se, and Alkali-W800-Se, were added to red soil or brown soil extracts [38,39]. Pseudo-first-order kinetics and pseudo-second-order kinetics models were used to fit the Se-releasing data on the first day. The release of four PEI-modified biochar Se fertilizers in both red soil and brown soil extracts were well fitted with pseudo-second-order kinetic fitting (Figure 7, Table 3). This indicated that the release of four PEI-modified biochar-based Se fertilizers in red soil or brown soil might be through the ion exchange reaction. The anion concentration (SO_4_^2−^, Cl^−^, and NO_3_^−^) in red soil extract was higher than that in brown soil extract (Table S4). This led to the equilibrium release amounts of Acid-W300-Se, Alkali-W300-Se, Acid-W800-Se, and Alkali-W800-Se in red soil extracts being all higher than those in brown soil extracts on the first day [30,37,38].

**Figure 8:**
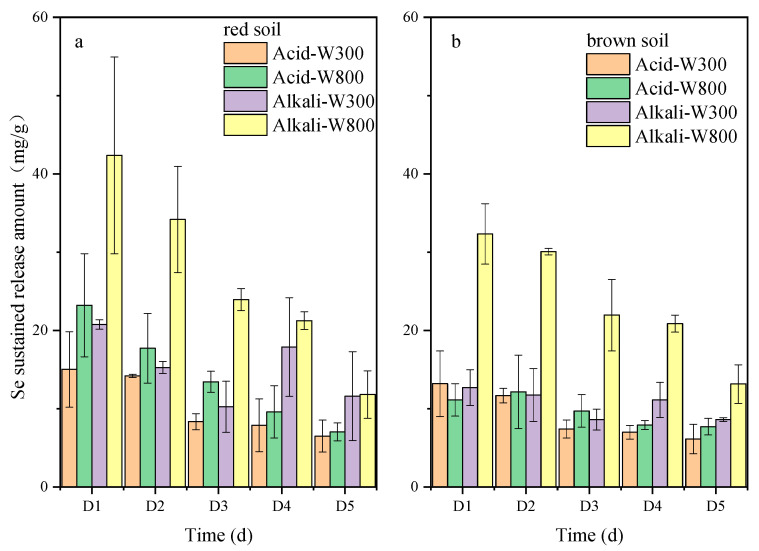
The daily Se release of Acid-W300-Se, Acid-W800-Se, Alkali-W300-Se, and Alkali-W800-Se in daily newly added red (**a**) and brown (**b**) soil solutions.

Additionally, the *k*_2*d*_ values of Alkali-W800-Se and Acid-W800-Se in the red soil extract were lower than those of Acid-W300-Se and Alkali-W300-Se, indicating that Alkali-W800-Se and Acid-W800-Se had higher release rates in red soil [38,39,40]. The equilibrium release of Alkali-W800-Se and Acid-W800-Se in the red soil extract was 42.34 mg/g and 32.33 mg/g on day 1, accounting for 13.48% and 9.36% of the total Se in Alkali-W800-Se and Acid-W800-Se, respectively (Figure 8, Table 3). The equilibrium release of Se via Acid-W300-Se and Alkaline-W300-Se in the red soil extract, however, was 15.01 mg/g and 23.21 mg/g, accounting for 5.23% and 7.44% of its total loading Se, respectively. After 5 days of continuous releasing, Alkali-W300-Se, Acid-W800-Se, and Alkali-W800-Se released 75.68 mg/g, 70.95 mg/g, and 133.47 mg/g of Se, accounting for 27.14%, 28.63%, and 42.49% of its total loading Se, respectively.

In brown soil, Alkali-W800 also showed similar characteristics, and its 5-day release in the brown soil extract was 118.33 mg/g, about 37.67% of its total loading Se. However, the Se release amounts of Acid-W300-Se for 5 days in red soil and brown soil were 83.43 mg/g and 72.87 mg/g, accounting for 18.07% and 15.79% of the total Se in Acid-W300-Se, respectively (Figure 8). As mentioned above, the Se in Alkali-W800-Se and Acid-W800-Se was mainly connected with amino groups through imine- and amide-bonding. The Se in those fertilizers was easily exchanged by anions in the soil solution. This led to the Se of Alkali-W800-Se and Acid-W800-Se being released quickly in the soil solution, which might cause the loss of Se [41,42,43]. In addition to the imine- and amide-bonding with selenium, a large part of selenium was through a chemical reaction with the C=N in Acid-W300 to form C-Se. This led to the Se release of Acid-W300-Se being controlled and slowed in red and brown soils. Studies showed that low-temperature pyrolysis biochar was more likely to age and decompose in the soil [13]. The Se in Acid-W300-Se will be continuously released with biochar aging to increase a crop’s Se content [42,44,45].

## 4. Conclusions

Our results demonstrated that both acid- and alkali-treated biochar could be cross-linked with PEI to increase the loading capacity of Se. The loading of SeO_3_^2−^ by four PEI-modified biochars was through imine- and amide-bonding and in the forms of a C-Se or N-Se bond. Alkali-W800-Se had the highest Se-loaded capacity (314.1 ± 1.6 mg/g) and the maximum Se exchangeable release rate (87.5%) compared with the other Se fertilizers. However, because the loading of Se in Alkali-W800-Se was mainly through imine- and amide-bonding, the Se release rate of Alkali-W800-Se was too fast to be used as a controlled and slow-release Se fertilizer in red and brown soils. The loading capacity of Se and the maximum Se exchangeable release rate in Acid-W300-Se were 287.1 mg/g and 62.2%, respectively. However, its slow and controllable release of Se makes Acid-W300-Se more suitable for use as a controlled and slow-release Se fertilizer in red soil and brown soil.

## Figures and Tables

**Table 1:** Elemental composition of biochar and PEI-modified biochar.

Samples *	C	H	O	N	S	H/C	O/C	(O + N)/C
	%	%	%	%	%			
W0	47.00	7.20	40.50	0.20	0.02	0.15	0.86	0.87
W300	67.90	5.40	25.80	0.30	0.15	0.08	0.38	0.38
W800	91.73	1.90	5.90	0.40	0.07	0.02	0.06	0.07
Acid-W300	49.16	8.40	30.70	11.7	0.04	0.17	0.62	0.86
Acid-W800	80.40	1.70	14.30	1.50	0.08	0.02	0.18	0.20
Alkali-W300	56.20	7.60	29.70	6.30	0.03	0.14	0.53	0.64
Alkali-W800	76.90	4.28	13.00	5.78	0.03	0.06	0.17	0.24

* The W0 presents the eucalyptus wood chips; W300 presents the biochar produced at 300 °C; W800 presents the biochar produced at 800 °C; Acid-W300 presents PEI-modified W300 after treated with HNO_3_; Acid-W800 presents PEI-modified W800 after treated withHNO_3_; Alkali-W300 presents PEI-modified W300 after treated with NaOH; and Alkali-W800 presents PEI-modified W800 after treated with NaOH.

**Table 2:** The load capacity, exchangeable release Se, and release rate on Acid-W300, Alkaline-W300, Acid-W800, and Alkaline-W800 *.

Biochar Selenium	Loading Capacity of Se	The Maximum Exchangeable Release of Se	Se-Exchangeable Release Rate
	mg/g	mg/g	%
Acid-W300-Se	287.1 ± 9.3 ab	178.6 ± 33.5 ab	62.2
Alkali-W300-Se	278.8 ± 17.6 ab	198.9 ± 29.4 ab	71.3
Acid-W800-Se	247.8 ± 4.9 b	176.7 ± 2.7 b	71.1
Alkali-W800-Se	314.1 ± 1.6 a	275.0 ± 29.5 a	87.5

* The different lowercase letters in the same column indicate a significant difference at *p* < 0.05 using ANOVA with Duncan’s test.

**Table 3:** Kinetic parameters of desorption of Acid-W300-Se, Acid-W800-Se, Alkali-W300-Se, and Alkali-W800-Se in extractions of red soil and brown soil.

Leaching Solution	Desorbent	PFOM			PSOM		
		*k*_1*d*_	*q_a_*	R^2^	*k*_2*d*_	*q_a_*	R^2^
Red soil extract	Acid-W300-Se	1.193	15.867	0.826	1.527	17.388	0.826
	Alkali-W300-Se	1.349	16.407	0.73	1.611	18.147	0.744
	Acid-W800-Se	1.057	16.469	0.646	1.203	18.559	0.692
	Alkali-W800-Se	1.104	36.838	0.771	1.214	41.487	0.76
Brown soil extract	Acid-W300-Se	1.309	13.455	0.903	1.716	14.61	0.855
	Alkali-W300-Se	0.787	16.423	0.722	1.032	18.202	0.741
	Acid-W800-Se	0.983	11.501	0.616	1.306	12.615	0.633
	Alkali-W800-Se	1.596	28.32	0.765	2.055	30.692	0.706

## Data Availability

Data are contained within the article.

## Supplementary Materials

The following supporting information can be downloaded at: https://www.mdpi.com/article/10.3390/ma17040879/s1, Figure S1: Scanning electron micrographs (SEM) of scanning electron micrographs of W300 (a), Acid-W300 (b), Alkali-W300 (c), W800 (d), Acid-W800 (e), and Alkali-W800 (f); Figure S2: The effect of Se initial concentration (a) and contact time (b) on Se loading amounts; Table S1: The Zeta potential of the biochar before and after modification; Table S2: Adsorption isothermal fitting parameters of Se adsorption onto Acid-W300, Acid-W800, Alkali-W300, and Alkali-W800; Table S3: Adsorption kinetic fitting parameters of Se adsorption onto Acid-W300, Acid-W800, Alkali-W300, and Alkali-W800; Table S4: The ion concentration and pH in red and brown soil extracts.
