# Supplementary material for: Effects of Pyrolysis Temperature and Acid-Base Pre-Treatment on the Synthesis of Biochar-Based Slow-Release Selenium Fertilizer and Its Release in Soil
Source: Materials (Basel). 2024 Feb 14;17(4):879. doi: 10.3390/ma17040879 (PMC10890299; doi:10.3390/ma17040879)
Supplement: Supplementary file 1 [file materials-17-00879-s001.zip › materials-2808619-supplementary.pdf]

Effects of Pyrolysis Temperature and Acid-Base Pre-Treatment on the Synthesis of Biochar-Based Slow-Release Selenium Fertilizer and Its Release in Soil

Table S1. The Zeta potential of the biochar before and after modification.

Samples	Zeta Potential
W300	-26.24±1.01mV
W800	-43.11±1.63mV
Acid-W300	27.12±1.02mV
Acid-W800	24.12±0.87mV
Alkali-W300	30.46±1.24mV
Alkali-W800	43.76±0.93mV

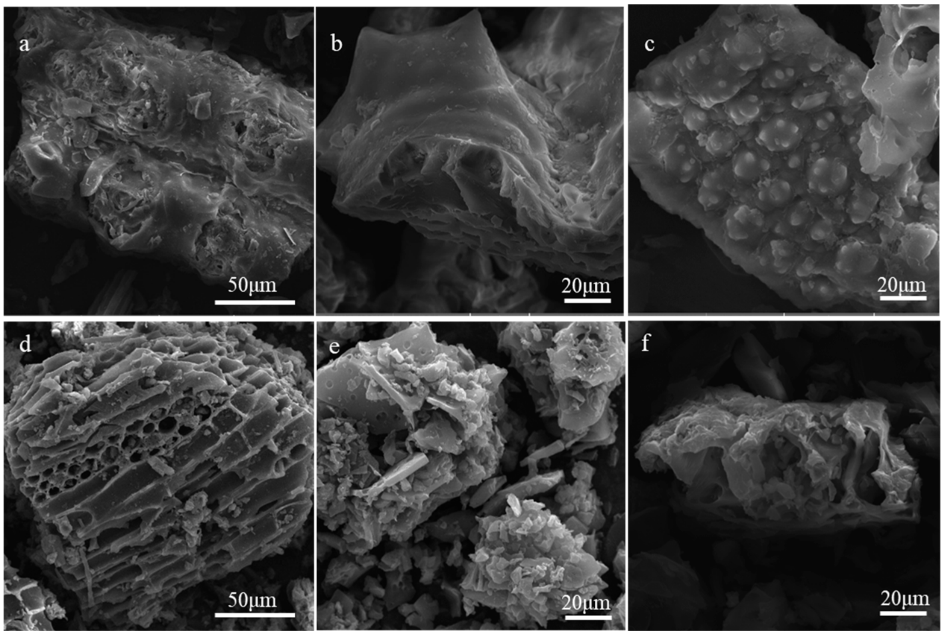

Figure S1. Scanning electron micrographs (SEM) of Scanning electron micrographs of W300(a), Acid-W300(b), Alkali-W300(c), W800(d), Acid-W800(e), and Alkali-W800(f) .

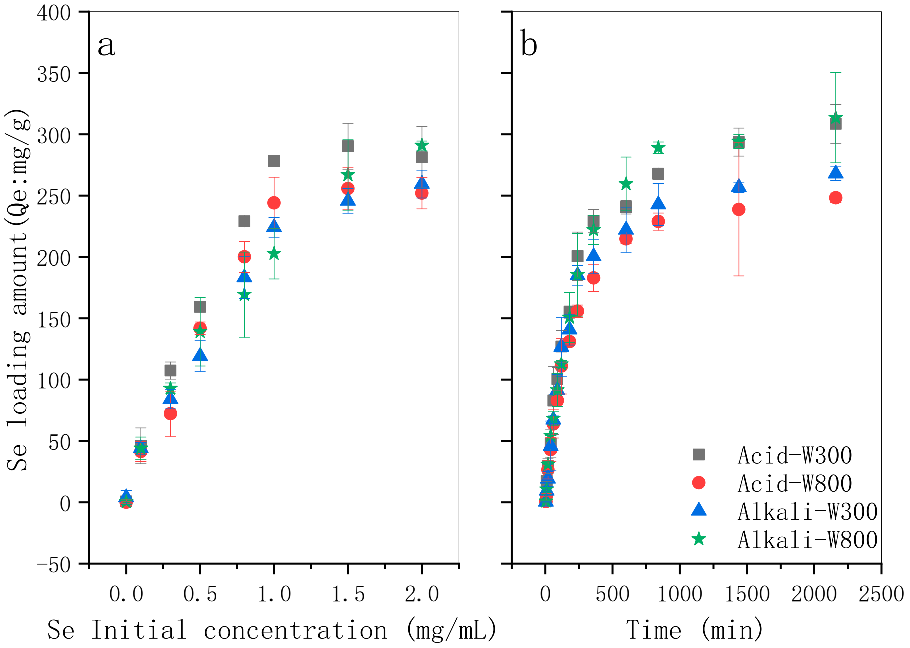

Figure S2. The effect of Se initial concentration (a) and contact time (b) on Se loading amounts.

Table S2. Adsorption isothermal fitting parameters of Se adsorption onto Acid-W300, Acid-W800, Alkali-W300, and Alkali-W800.

Isothermal adsorption model	Model parameters	Acid-W300	Acid-W800	Alkali-W300	Alkali-W800
Langmuir	K	9.667	4.926	3.758	3.616
	Qmax	329.156	315.041	321.927	344.332
	R^2	0.896	0.898	0.932	0.904
Freundlich	K_f	304.658	255.593	251.158	272.134
	n	0.243	0.303	0.358	0.384
	R^2	0.916	0.847	0.915	0.96
Temkin	b	45.884	36.483	36.067	37.168
	A	249.852	47.815	39.598	53.264
	R^2	0.931	0.886	0.938	0.947

Table S3. Adsorption kinetic fitting parameters of Se adsorption onto Acid-W300, Acid-W800, Alkali-W300, and Alkali-W800.

Adsorbents	q_e(exp)	PFOM		PSOM		Elovich		
		k_1	R^2	k_2	R^2	α	β	R^2
**Acid-W300**	308.53	0.278	0.986	0.401	0.994	0.394	5.741	0.977
Acid-W800	248.21	0.275	0.994	0.396	0.997	0.407	5.640	0.974
Alkali-W300	267.90	0.286	0.991	0.413	0.995	0.412	5.701	0.972
Alkali-W800	313.52	0.289	0.988	0.420	0.995	0.412	5.753	0.974

Table S4. The ion concentration and pH in red and brown soil extract.

Soil extract	pH	SO_4^{2-}	Cl^{-1}	PO_4^{3-}	NO_3^-	CO_3^{2-}	SeO_3^{2-}
		mg/L	mg/L	mg/L	mg/L	cmol/L	mg/L
Red soil extract	5.53±0.02	4.33±0.08	5.65±0.12	/	15.90±1. 12	/	/
Brown soil extract	4.81±0.01	2.91±0.04	1.70±0.09	/	12.30±0. 95	/	/
